# Spontaneous perforation of Meckel’s diverticulum in an adult female with literature review

**DOI:** 10.1186/s40792-018-0536-y

**Published:** 2018-11-01

**Authors:** Andrew A. Fraser, Douglas D. Opie, James Gnecco, Beshoy Nashed, David C. Johnson

**Affiliations:** 1 General Surgery Department, Mountain Vista Medical Center, 1301 S Crismon, Mesa, AZ 85209 USA; 2 Glendale, USA; 3 Mesa, USA

**Keywords:** Meckel’s diverticulum, Perforation, Diagnosis, Resection

## Abstract

**Background:**

Perforated Meckel’s diverticulum is a rare complication of an already rare disease process, which often mimics a perforated appendix on presentation and diagnosis. The majority of case reports for perforation involve either a foreign body or fecalith.

**Case presentation:**

We report the case of a 54-year-old female who initially presented at another institution with signs and symptoms of acute appendicitis and underwent appendectomy with a drain left in place. Subsequently, she underwent exploratory laparotomy at our institution for a perceived stump leak or incidental perforation and was found to have a perforated Meckel’s, with no evidence of foreign body or fecalith.

**Conclusion:**

The literature of Meckel’s diverticulum is reviewed, and we discuss the difficulty in diagnosis as well as the quandary of incidental resection.

## Background/introduction

The father of German surgery, Wilhelm Fabry (Fabricius Hildanus), was the first to describe ileal diverticula in 1598. These diverticula were later characterized as branches of a tree and found within a strangulated hernia by the French surgeon and anatomist, Alexis Littre, in 1700 [1]. It was not until 1809, however, that a fellow German comparative anatomist, Johann Friedrich Meckel, described the embryonic origin, and thus, a fundamental understanding of the diverticulum that now bears his name [2].

Meckel’s diverticulum (MD) is the result of the incomplete obliteration of the vitelline ducts. It is often referred to by the rule of 2’s: 2% of the population, within 2 ft of the ileocecal valve, 2 in. in length, 2 types of heterotopic mucosa, and presentation before the age of 2. MD is the most common congenital abnormality of the gastrointestinal tract; however, it rarely presents symptomatically. The most common presenting symptom of MD is gastrointestinal bleeding in children; while in adults, the most common presentation is obstruction, either due to intussusception or adhesive bands [3, 4]. In both populations, perforation is an uncommon presentation, particularly among older aged women. Perforated MD often presents as does perforated acute appendicitis; namely with fever, chills, nausea, vomiting, right lower quadrant abdominal pain, and peritoneal signs. This semblance often results in diagnostic inaccuracy. Despite the uncommon incidence of symptomatic MD in adults, the morbidity associated with adult symptomatic MD is significant. The available research, however, remains inconclusive on whether or not an incidental MD should be prophylactically excised.

We present the case of an otherwise healthy 54-year-old female who presented with a clinical diagnosis of acute appendicitis and a normal CT scan. She was initially treated with diagnostic laparoscopy and appendectomy, but was subsequently diagnosed with perforated Meckel’s diverticulum after her condition worsened and underwent exploratory laparotomy.

## Case presentation

A 54-year-old Caucasian female with a history of obstructive sleep apnea presented to her regional hospital with symptoms of fever, nausea, and right lower quadrant abdominal pain. She was clinically diagnosed with acute appendicitis; however, CT scan showed a normal appendix, with no other acute abdominal findings. The decision was made to proceed with diagnostic laparoscopy. Extensive inflammatory adhesions were found in the RLQ, with an appendix that appeared acutely inflamed with surrounding phlegmon. The appendix was removed, and a Jackson-Pratt drain was left to drain any residual infection. The patient initially improved and was discharged home on the second postoperative day. Surgical pathology revealed acute appendiceal serositis with a rare small focus of mucosal inflammation and no evidence of perforation. A few days after discharge, the patient’s JP drainage increased and began to be feculent in appearance. Her symptoms of nausea, fever, and chills returned, with persistent abdominal pain and constipation.

The patient returned to the hospital due to her worsening symptoms and was transferred to our facility for continued care. Upon presentation at our facility, she was slightly febrile and hypertensive, with no tachycardia or tachypnea. Lab work revealed a white cell count of 21,500 and lactic acid of 1.0. On physical exam, she was 5 ft. and 6 in. tall and weighed 316 lbs. with a BMI of 51. She had significant lower quadrant tenderness to palpation and had developed substantial cellulitic changes around the drain site, with dark, feculent drainage within, and around the drain.

**Fig. 1:**
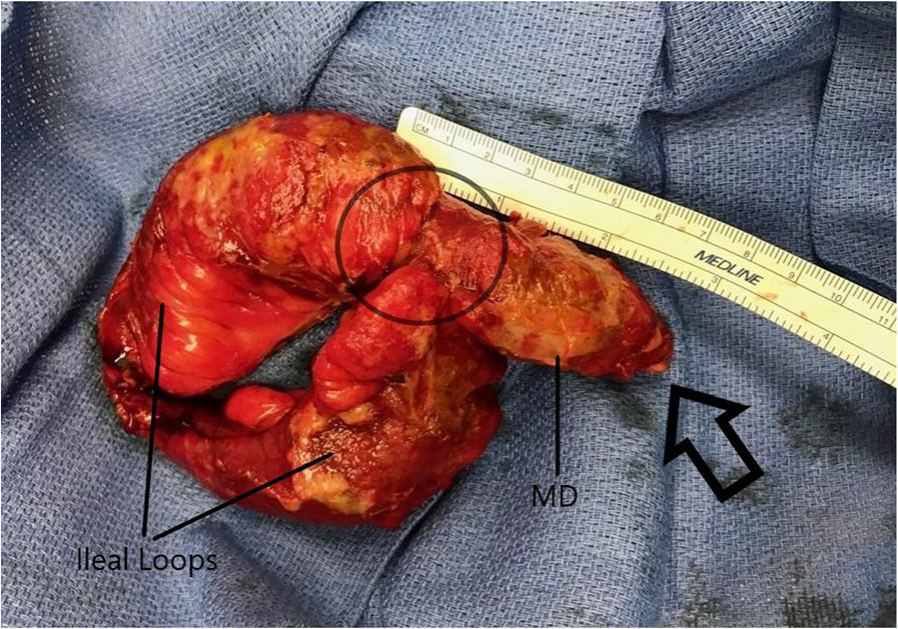
Five centimeters of Meckel’s diverticula along a segment of ilium. Note the perforation at the apex (arrow) and narrow base (circle). Histology revealed evidence of pressure necrosis at the apex with perforation with no evidence of ectopic tissue or malignancy (data not shown)

A diagnosis of appendiceal stump dehiscence vs bowel perforation was made, and the patient was taken to the operating room for an exploratory laparotomy. Upon entering the abdomen, copious amounts of enteric contents were found, with significant inflammation throughout. The cecum appeared intact, with no evidence of staple line dehiscence. A systematic examination of the viscera revealed an ileal diverticulum approximately 55 cm from the ileocecal valve, with perforation at its apex (Fig. 1). A wedge resection of the diverticulum containing ileum with primary anastomosis was performed. The abdomen was washed out and closed, and a new JP drain was placed.

Extracorporeal inspection of the specimen revealed a 5-cm-long diverticulum with a narrow base. No evidence of foreign body or fecalith was found either within the diverticula or during abdominal washout and repeat inspection. Histology revealed evidence of pressure necrosis at the apex with perforation. No evidence of ectopic tissue or malignancy.

Postoperatively, the patient was managed in the intensive care unit. Her course was complicated with dehiscence of the ileo-ileal anastomosis. She required a second wash out and an end ileostomy, after which, her condition improved. She was discharged on postoperative day 16 from her diverticulum resection. She underwent ileostomy takedown 3 months later.

## Discussion/conclusion

Meckel’s diverticulum is the result of dysgenesis of the small bowel as it develops in relation to the embryologic yolk sack. It arises from the incomplete obliteration of the vitelline duct between the 5th and 8th weeks of gestation. As such, MD is a true diverticulum, with its own blood supply. Ectopic tissue is found in up to 60% of symptomatic cases, with gastric mucosa most frequently discovered [5].

Meckel’s diverticulum is the most common congenital malformation of the gastrointestinal tract; yet, it only affects between 2 and 4% of the population. Manifesting as gastrointestinal bleeding, symptomatic MD is more commonly seen in children, with a mean age of presentation at age 10 [6]. Among adults, symptomatic MD is even rarer. The estimated lifetime risk for developing complications of MD range from 4 to 6.4% [7, 8].

The most common presenting symptom in adults is bowel obstruction, followed by GI bleed and lastly, diverticulitis [3, 4]. Some studies estimate the incidence of diverticulitis to be 20% of symptomatic cases [5, 9]. Spontaneous perforation due to diverticulitis is even less common, especially in older females [10–14]. A study by Chen et al. concerning the behavior of MD in children showed only 7.3% of symptomatic MD was perforated [15]. Meanwhile, the Mayo Clinic reported 10–12% of symptomatic patients with perforation [16].

Accurate diagnosis in symptomatic MD has only been estimated between 5.7 and 13% (Table 1), with symptomatic MD reported to be 55× rarer than acute appendicitis [6]. In an investigation of 600 MD patients, less than 50% were taken to the OR with a specific preoperative diagnosis, and only 5.7% of them were preoperatively diagnosed with MD [17]. In another study, 57.5% of patients were initially misdiagnosed, including 21.6% of patients misdiagnosed as appendicitis [15]. This study also included six patients who underwent appendectomy at another institution, and were subsequently transferred, and found to have a missed diagnosis of complicated MD [15]. In a study of perforated MD in adults, Ding et al. found that 60% of patients were diagnosed with perforated appendicitis preoperatively, while only 13% were diagnosed with perforated MD [9]. This may be due to the fact that Meckel’s diverticulitis is often symptomatically indistinguishable from appendicitis, as the disease process is identical.

**Table 1:** Estimated rates of accurate diagnosis

Citation	Patients reported	Number misdiagnosed	Estimated accurate diagnosis rate (%)
Yamaguchi et al. (1978) [17]	600 (287)*	566	5.7
Kusumoto et al. (1992) [24]	274	29	10.6
Bani-Hani et al. (2004) [20]	68	64	5.9
Sai et al. (2007) [25]	36	20**	44
Ding et al. (2012) [9]	15	13	13
Chen et al. (2018) [15]	233	134**	42.49

*Only 287 of the 600 patients had a preoperative diagnosis before surgery

**The remainder of children presented with GI bleed; thus, a misdiagnosis of MD with symptoms other than GI bleed approached 100%

The difficulty of preoperatively distinguishing appendicitis and Meckel’s diverticulitis is compounded by the inadequacy of imaging studies. MD is usually indistinguishable from normal loops of small bowel on CT scan; meanwhile, a nonvisualized or normal-looking appendix on CT does not necessarily rule out appendicitis. A retrospective analysis by Baldisserotto et al. evaluated the use of ultrasound in diagnosing MD. In the study, the diverticula were visualized in 9 out of 10 patients, and yet, 9 out of the 10 cases were misdiagnosed as appendicitis or obstruction, with only one case confirmed by scintigraphy, which was performed based on symptomatology [18]. This further solidifies the indistinguishable nature of MD from normal anatomy or abnormal pathology in most imaging modalities.

Radionuclide scans (Meckel’s scans) are the most promising imaging modality for diagnosing MD. However, this only seems to be applicable in the pediatric population. Scintigraphy has upwards of 90% accuracy in the pediatric population, but its usefulness drops to 46% accuracy in the adult population [19]. As a result of radionuclide inaccuracy, as well as the previously mentioned diagnostic difficulties, less than 10% of MD are diagnosed preoperatively in adults [17, 20].

With such difficulty in diagnosing MD preoperatively, the majority of MD are diagnosed during surgery, usually after the initial diagnosis of appendicitis or other abnormality has been ruled out [21, 17]. That being said, it is worth mentioning that one institution revealed that no search for MD was reported in 19.1% of their 9793 appendectomies [22], indicating that although MD should be considered within the differential for appendicitis, it may often be overlooked even in the operating room.

The question of what to do with an incidentally discovered MD is still a controversial subject. The majority of literature on MD cites Soltero and Bill [8] in 1976 for a decreasing incidence of MD complications with age, approaching 0% risk by the age of 75. This forms the basis for the current recommendations by most major texts for leaving an incidentally discovered MD in situ. However, the more recent population-based epidemiological study performed by Cullen et al. [7] finds no decrease in the risk of complications throughout life, with an associated lifetime risk of 6.4%. Thus, contrary to conventional surgical dogma, they advocate for the removal of incidental MD. Other studies have reported the need of surgical intervention for complicated MD in patients up to 91 years of age [4, 16], and Sagar et al. further bolster this notion. They found morbidity rates as high as 33% after resection of complicated MD [4]. Interestingly enough, the most recent publication by Zani et al. found similar results to Cullen in that the risk of death did not change significantly with age. In fact, despite there being more diverticulectomies performed in the pediatric population, the majority (94%) of deaths from MD were in adults older than age 44 [23]. Notwithstanding these results, they came to the conclusion that incidental MD should be left alone (Table 2); basing their recommendations on their calculations for resection complication rates, number needed to treat, and their analysis of long-term follow-up with patients where the MD was left in place.

**Table 2:** Resection recommendations for incidental findings

Citation	Date of study	Number of patients	Estimated lifetime risk of complications (%)	Recommendations
Soltero et al. [8]	1976	202	4*	Do not resect
Cullen et al. [7]	1994	58	6.4	resect
Zani et al. [23]	2008	+	4	Do not resect
Park et al. [16]	2005	1476	16**	Selective resection

*This risk decreases to 0% by the age of 76

**This percentage is number of symptomatic patients

+This study involved a systematic review of the literature and various population-based data

This does raise a good question, however: What is the rate of complicated MD among those in whom an incidental Meckel’s was left in place? Zani et al. analyzed five studies for a long-term follow-up of known MD with only 101 patients between them and varying years, between 2 and 54, of follow-up. One study analyzed, attempted to follow 45 patients who had an incidental MD left in place. However, they had a significant rate of attrition greater than 50%, and therefore, their results were not statistically significant [22]. Meanwhile, other studies seem to contradict Zani’s findings. Kusumoto et al. found that upwards of 63% of symptomatic patients over the age of 20, who required surgical intervention, had previous symptoms attributable to MD [24]. In yet another study by Bani-Hani and Shatnawi, 4 patients (14.3%) out of 28 with diverticulum related complications had a known history of MD [20]. If the complication rate among patients with a known history of MD is indeed significant, it would seem prudent to recommend prophylactic removal of incidental diverticula. Definitive conclusions certainly cannot be made from case reports such as this one presented; nevertheless, based on the available research, and our own experience, it is our opinion that prophylactic removal of incidental diverticula is advisable.

Our patient is a 54-year-old female who sustained significant morbidity due to her complicated MD. Her case is interesting in that she does not fit the common presentation of MD, due to her age and sex. She is also noteworthy due to the nature of her complication of spontaneous perforation. Based on a review of the relevant literature, perhaps further examination of the complication rates among known MD would be beneficial in answering the question: should incidental MD be removed prophylactically or not?

## Abbreviation

MD: Meckel’s diverticula
